# Age-specific effects of ozone on pneumonia in Korean children and adolescents: a nationwide time-series study

**DOI:** 10.4178/epih.e2022002

**Published:** 2021-12-28

**Authors:** Kyoung-Nam Kim, Youn-Hee Lim, Sanghyuk Bae, In Gyu Song, Soontae Kim, Yun-Chul Hong

**Affiliations:** 1 Department of Preventive Medicine and Public Health, Ajou University School of Medicine, Suwon, Korea; 2 Department of Public Health, University of Copenhagen, Copenhagen, Denmark; 3 Department of Preventive Medicine, College of Medicine, The Catholic University of Korea, Seoul, Korea; 4 Department of Pediatrics, Korea University Guro Hospital, Seoul, Korea; 5 Department of Environmental and Safety Engineering, Ajou University, Suwon, Korea; 6 Department of Preventive Medicine, Seoul National University College of Medicine, Seoul, Korea; 7 Institute of Public Health and Medical Care, Seoul National University Hospital, Seoul, Korea; 8 Institute of Environmental Medicine, Seoul National University Medical Research Center, Seoul, Korea

**Keywords:** Ozone, Pneumonia, Children, Adolescent, Time-series analysis

## Abstract

**OBJECTIVES:**

The aim of this study was to estimate the age-specific effects of 8-hour maximum ozone levels on pneumonia in children and adolescents.

**METHODS:**

We performed quasi-Poisson regression analyses for individuals of 0-4 years, 5-9 years, 10-14 years, and 15-19 years of age using nationwide time-series data from the Korea (2011-2015). We constructed distributed lag linear models employing a generalized difference-in-differences method and controlling for other air pollutants.

**RESULTS:**

A 10.0-parts per billion increase in 8-hour maximum ozone levels was associated with a higher risk of hospital admissions due to pneumonia at 0-4 (relative risk [RR], 1.02; 95% confidence interval [CI], 1.01 to 1.03) and 5-9 years of age (RR, 1.06; 95% CI, 1.04 to 1.08), but not at 10-14 (RR, 1.01; 95% CI, 0.98 to 1.04) or 15-19 years of age (RR, 1.01; 95% CI, 0.97 to 1.06). The association between ozone and hospital admissions due to pneumonia was stronger in cool seasons (from November to April) than in warm seasons (from May to October), but was similar between boys and girls.

**CONCLUSIONS:**

Short-term exposure to ozone was associated with a higher risk of pneumonia at 0-4 years and 5-9 years of age, but not at 10-14 years or 15-19 years of age. Our findings can help identify vulnerable periods, determine the target populations for public health interventions, and establish air pollution standards.

## GRAPHICAL ABSTRACT

[Fig f2-epih-44-e2022002]

## INTRODUCTION

Ambient ozone, an air pollutant, is a global public health issue. Ozone has strong oxidative potential and has been associated with various health outcomes, such as airway inflammation [1], decreased lung function [2], hospital admission due to respiratory diseases [3], and mortality [4]. Because ozone is primarily generated from photochemical reactions of other air pollutants under high temperatures and sunlight, ozone levels are predicted to rise globally due to climate change [5].

Previous experimental studies have suggested that ozone can increase the susceptibility to respiratory infections, such as pneumonia [6,7]. However, the number of epidemiological studies on the association between ozone and pneumonia is insufficient compared with that of studies investigating the association between other pollutants (e.g., particulate matter with an aerodynamic diameter ≤ 2.5 µm [PM_2.5_]) and pneumonia, and their results are inconsistent [8-15]. For example, short-term exposure to ozone has been associated with a higher risk of pneumonia in time-series studies conducted in the United States [9] and China [8], while an association was not found in a case-crossover study in the United States [10]. In addition, although previous studies have suggested that there may be an effect modification by age on the association between ozone and pneumonia [8,9], and that children and adolescents may be especially susceptible to air pollution [16], evidence for these younger populations is limited [9,11-14].

Therefore, using nationwide time-series data of Korean children and adolescents, we investigated the association between ozone exposure and pneumonia, which represents the most common cause of hospitalization among individuals under 18 years of age [17] and the second most expensive condition for hospitalization among children [18]. We hypothesized that the effect size would be larger among younger than older subjects, and we therefore performed analyses stratified by age group and compared the results in each stratum.

## MATERIALS AND METHODS

### Patient and hospital admission data

We performed a time-series study using data on daily counts of hospital admissions with a principal admission diagnosis of pneumonia (International Classification of Diseases, 10th revision code J12-J18) among children and adolescents (0-19 years of age) between January 1, 2011 and December 31, 2015. We obtained data from the National Health Insurance Service (NHIS) of Korea, which provides universal health coverage to all residents of Korea [19]. In this study, the NHIS generated and provided data by aggregating the count of only the first day of admission for each hospitalization. The accuracy of diagnoses in the NHIS data has been previously reported to be high, especially for inpatient settings [20-23]. The outcome daily counts were aggregated by region (16 regions constituting of Korea) (Supplementary Material 1), age group (0-4, 5-9, 10-14, and 15-19 years), and sex, leading to 128 separate timeseries data sets.

### Air pollution and meteorological data

We estimated 8-hour maximum ozone concentrations (highest 8-hour moving average concentration) and daily mean concentrations of particulate matter with an aerodynamic diameter ≤ 10 µm (PM_10_), nitrogen dioxide (NO_2_), sulfur dioxide (SO_2_), and carbon monoxide (CO) in each region from the 24-hour monitoring data of urban monitoring stations. We obtained this data from the National Ambient Air Monitoring Information System. This national system continuously collects data from 318 fixed-site monitoring stations, which are located based on the population distribution and operated under strict quality control [24]. We first calculated 8-hour maximum ozone levels and daily mean PM_10_, NO_2_, SO_2_, and CO levels at each monitoring station after excluding missing values (< 5% of total data). We then calculated region-specific values by averaging 8-hour maximum ozone levels and daily mean PM_10_, NO_2_, SO_2_, and CO levels from all monitoring stations located in each region. Because nationwide monitoring of PM_2.5_ was not conducted during the study period (2011-2015), we did not consider PM_2.5_ levels in the present study.

We estimated daily mean temperature (°C) and relative humidity (%) for each region using 24-hour measurement data from the Korea National Meteorological Administration. If there were multiple weather stations in a region, we used average temperature and relative humidity values for further analyses.

### Statistical analysis

We performed quasi-Poisson generalized linear regression analyses to evaluate the short-term effects of 8-hour maximum ozone levels on hospital admissions due to pneumonia. All further analyses were performed using data stratified by the age groups of 0-4 years, 5-9 years, 10-14 years, and 15-19 years.

To assess the delayed and cumulative effects of lag-day exposures, we constructed distributed lag linear models [25]. In these models, we implemented lag structures up to 7 days with a polynomial function with 4 degrees of freedom, according to previous studies investigating the association between short-term exposure to ozone levels and respiratory infection-related outcomes [9,11] and the lag-specific associations between ozone and pneumonia estimated from distributed lag linear models (adjusted for region, day, temperature, relative humidity, population, and other air pollutants) (Supplementary Material 2). We selected the degrees of freedom according to a previous study [26]. However, we also confirmed the robustness of the results in models with different degrees of freedom in sensitivity analyses.

To improve causal inference by adjusting for confounders related to spatial units of the analysis (i.e., regions) and those related to temporal units (i.e., days), we employed a generalized differencein-differences method [27,28], a formal causal modeling approach [27], by introducing dummy variables for spatial units (16 regions) and temporal units (1,826 days) in the time-series analytical models. The common form of the difference-in-differences method leverages 2 areas (or exposures) and 2 time points. However, the difference-in-differences method can be applied to data with multiple spatial and temporal units by examining the association between the exposure and outcome of interest (both measured at the level of a predefined spatial and temporal unit) adjusted for spatial and temporal units [29,30]; therefore, in the context of short-term air pollution epidemiology, it can be applied as a multi-region time-series study adjusted for spatial (e.g., regions) and temporal units (e.g., days). This method can control for both measured and unmeasured confounders, such as socio-demographic factors [27,28]. Therefore, although this method is computationally demanding, it has an edge over other causal inference methods, such as those using a propensity score, which can only control for measured confounders.

We also adjusted for daily mean temperature and relative humidity up to 7 days, all of which were modeled with a natural cubic spline with 3 degrees of freedom according to previous studies [8,11], and the log-transformed population of each region as an offset. Finally, to confirm that the results were not confounded by other air pollutants, we further adjusted the analyses for daily mean PM_10_, NO_2_, SO_2_, and CO levels up to 7 days. Therefore, the main analytical models from which we extracted the main findings can be summarized as follows:


$$\log[E(Y_{s,t})]=\beta_{0}+\beta_{1}Ozone_{s,t}+\beta_{2}D_{s}+\beta_{3}D_{t}+\beta_{4}Temp_{s,t}+\beta_{5}Humi_{s,t}+\beta_{6}Pollut_{s,t}+log(Pop_{s,t})$$

where Y_*s,t*_ is the number of hospital admissions due to pneumonia in region *s* on day *t*; Ozone_*s,t*_, Temp_*s,t*_, Humi_*s,t*_, and Pollut_*s,t*_ are the cross-basis matrices of ozone, temperature, relative humidity, and other air pollutants (PM_10_, NO_2_, SO_2_, and CO), respectively, in region *s* on day *t* with the aforementioned lag structures and concentration-response curves; D_*s*_ and D_*t*_ are dummy variables for regions and days, respectively; and Pops,t is the population of region s in the year of day t. All variance inflation factors estimated from quasi-Poisson regression models including terms for daily 8-hour maximum ozone levels and daily mean levels of PM_10_, NO_2_, SO_2_, CO, temperature, and relative humidity were lower than 4.0 in the age groups of 0-4 years, 5-9 years, 10-14 years, and 15-19 years of age. In addition, the shapes of the concentration-response curves for the associations between ozone and pneumonia at 0-4 years, 5-9 years, 10-14 years, and 15-19 years of age were investigated using distributed lag non-linear models adjusted for the same covariates (the concentration-response curves were modeled with a natural cubic spline with 3 degrees of freedom).

Because several studies have suggested heterogeneity of the association between ozone levels and pneumonia by seasons [8,10], we stratified the analyses according to warm seasons (from May to October) and cool seasons (from November to April). We also performed sex-stratified analyses to assess the associations’ similarity between boys and girls.

We conducted the following sensitivity analyses: First, we repeated the analyses after excluding the region with the lowest ozone levels (Seoul, 28.1 parts per billion [ppb]) and the one with the highest levels (Jeju Province, 41.6 ppb), to verify possible violations of the positivity assumption [31]. The positivity assumption, which is essential for causal inference, postulates that each exposure level (or intervention and control) could be observable at each observation. In this study, it was likely that some ozone levels would not be observed in the regions with the highest and lowest ozone levels, in which case there was a concern that causal inference could not be adequately made in these regions. Second, according to previous studies [9-12], we further adjusted for influenza epidemics, defined as a daily number of hospital admissions due to influenza of more than 80 cases in each region (1.0% of the study period). Third, we used different degrees of freedom for the lag structures (2 to 5 degrees of freedom) and concentration-response curves (2 to 5 degrees of freedom) in the distributed lag linear models. Fourth, we conducted conventional 2-stage time-series analyses to confirm the robustness of the results. In these analyses, we first obtained region-specific associations between ozone levels and pneumonia adjusted for calendar time (natural cubic spline, 8 degrees of freedom per year), daily mean temperature and relative humidity up to 7 days (cross-basis matrices, 3 degrees of freedom), and other pollutants (PM_10_, NO_2_, SO_2_, and CO) up to 7 days (cross-basis matrices). We then pooled the region-specific results by performing meta-analyses.

We conducted the analyses using R version 4.0.3 (R Foundation for Statistical Computing, Vienna, Austria).

### Ethics statement

Because we used secondary count data de-identified by the NHIS, the need for written informed consent was waived. The Ethics Review Board of Seoul National University Hospital approved the study protocol (approval No. E-1909-096-1065), and we performed the study according to the tenets of the Declaration of Helsinki.

## RESULTS

The mean± standard deviation daily counts of hospital admissions due to pneumonia during the study period (2011-2015) were 493± 276 at 0-4 years of age, 100± 90 at 5-9 years of age, 29± 26 at 10-14 years of age, and 12± 10 at 15-19 years of age (Table 1). The daily counts of hospital admissions due to pneumonia were higher in cool than in warm seasons (Supplementary Material 3). The mean 7-day moving average of the daily 8-hour maximum ozone levels during the study period was 37.3 ppb, which is comparable to the levels in the United States and other developed countries (Table 1) [32]. The ozone levels were higher in warm than in cool seasons, while PM_10_, NO_2_, SO_2_, and CO levels were higher in cool than in warm seasons (Supplementary Material 3).

The correlations of ozone levels with other air pollutant levels and meteorological factors were weak, with the absolute values of Spearman correlation coefficients less than 0.30, except for the correlation between ozone levels and temperature (r= 0.43, p< 0.001) (Supplementary Material 4).

**Figure 1.**
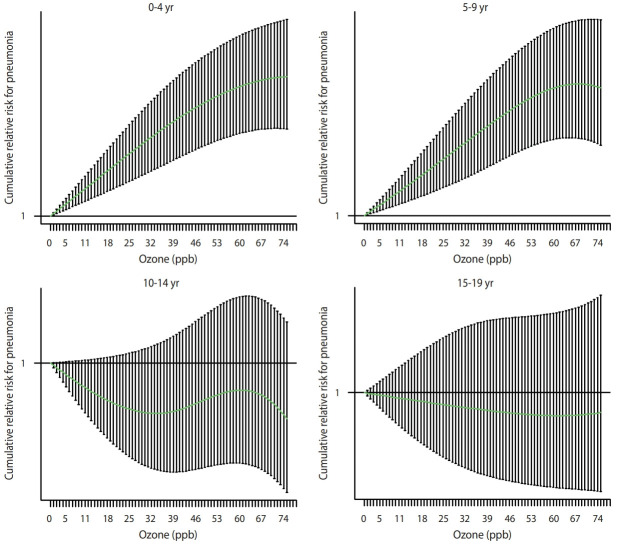
Concentration-response curves for the associations between ozone and pneumonia by age group, estimated from the distributed lag non-linear models adjusted for region, day, temperature, relative humidity, population, and other air pollutants (particulate matter with an aerodynamic diameter ≤10 μm, nitrogen dioxide, sulfur dioxide, and carbon monoxide). ppb, parts per billion.

After a visual inspection of the concentration-response curves for the associations between ozone levels and pneumonia (Figure 1), we estimated the delayed and cumulative effects of ozone levels up to 7 days on pneumonia using the distributed lag linear models. A 10.0-ppb increase in ozone levels was associated with a higher risk of pneumonia at 0-4 (relative risk [RR], 1.02; 95% confidence interval [CI], 1.01 to 1.03) and 5-9 years of age (RR, 1.06; 95% CI, 1.04 to 1.08), but not at 10-14 (RR, 1.01; 95% CI, 0.98 to 1.04) or 15-19 years of age (RR, 1.01; 95% CI, 0.97 to 1.06) (Table 2).

Using the same analytical models, we also found positive associations of a 10.0-µg/m^3^ increase in PM_10_ (RR, 1.01; 95% CI, 1.00 to 1.02) and a 1.0-ppb increase in SO_2_ (RR, 1.01; 95% CI: 1.00 to 1.01) with pneumonia at 0-4 years of age; however, in other age groups, the associations between air pollutant levels and pneumonia were generally null or inverse, except for the association with CO (Supplementary Material 5).

When we stratified the analyses by season, the association between ozone and pneumonia was stronger in cool seasons (from November to April) than in warm seasons (from May to October) (Table 2). However, we found no appreciable sex differences in the association between ozone and pneumonia (Supplementary Material 6).

We conducted sensitivity analyses using alternative data, covariate selections, and model specifications. First, the results were insensitive to the exclusion of the region with the lowest ozone levels (Supplementary Material 7) and the region with the highest ozone levels (Supplementary Material 8). Second, after further adjusting for influenza epidemics, the results did not change appreciably (Supplementary Material 9). Third, we found robust results using different degrees of freedom for the lag structures and concentration-response curves (data not shown). Fourth, the results were consistent between conventional 2-stage time-series analyses and the analyses applying the difference-in-differences method, except that the association at 5-9 years of age was only found in the difference-in-differences approach. This heterogeneity might be due to strict control of potential confounders (related to spatial and temporal units) by the difference-in-differences method (Supplementary Material 10).

## DISCUSSION

The ozone levels in Korea, as presented in this study, were comparable to those in the United States and other developed countries. We found that ozone levels were associated with a higher risk of hospital admissions due to pneumonia at 0-4 years and 5-9 years of age, but not at 10-14 years or 15-19 years of age, in the formal causal inference models. Because the socioeconomic burden of pneumonia in children and adolescents is high [17,18] and air pollution may increase the associated costs [33], the results of the present study have substantial public health implications.

Previous studies exploring the association between ozone exposure and pneumonia among children and adolescents are relatively rare and have yielded heterogeneous results [12]. For example, a time-series study conducted in 17 states of the United States reported a positive association between ozone levels and emergency department visits due to pneumonia at 0-18 years of age [9], while another time-series study in Hanoi, Vietnam did not find an association between ozone levels and hospital admissions due to pneumonia at 0-17 years of age [11]. Furthermore, few studies have identified high-risk age groups by stratifying children and adolescents by age: Some studies suggested that associations between ozone and pneumonia might be more prominent at 0-4 years of age than at older ages [13,14]. However, another time-series study failed to identify an association between ozone and pneumonia at 0-1 years and 1-5 years of age [11]. For older children and adolescents, a case-crossover study in California found no association between ozone levels and emergency department visits due to pneumonia at 5-18 years of age [13]. This observed heterogeneity may be attributable to differences in population characteristics (related, for example, to sensitivity and adaptive capacity), exposure levels, measurement error for exposure and outcome, and analytical strategies (e.g., study design, statistical models, and methods used to control for the confounding effects of various factors, including other air pollutants).

In the generalized difference-in-differences analyses, considering the effects of other air pollutants, we found an association between ozone levels and pneumonia at 0-4 years and 5-9 years of age, but not at 10-14 years or 15-19 years of age. It has been suggested that ozone exposure can increase the risk of airway infections by affecting host immunity [34,35]. Thus, the immature immune system of younger children may explain these findings [36]. The results might also reflect lower rates of outdoor activity (and time spent outdoors) among middle-school and high-school students compared with those of younger children in Korea [37], given that indoor ozone levels are generally lower than outdoor levels [15].

Most previous studies [8,10,11,13,15], but not all of them [14], have reported a stronger association between ozone and pneumonia in warm than in cool seasons. These findings are inconsistent with the results of this study, which showed a stronger association in cool seasons. The reason for this inconsistency is not clear. However, although ambient ozone levels are lower in cool seasons, outdoor activities are likely to increase on sunny days with higher temperatures (possibly days with higher ozone levels as well) in cool seasons but decrease on similar days in warm seasons, resulting in different individual exposure patterns according to seasons [14]. In addition, air conditioning with low ventilation due to closed windows in warm seasons (especially on sunny days with higher temperatures) can also prevent the penetration of outdoor ozone, hence reducing exposure [15]. Finally, the observed stronger association between ozone and pneumonia in cool seasons might be affected by seasonal differences in ozone levels and their variance (Supplementary Material 3) or the unmeasured confounding effect of seasonal variations in infections. However, we found robust results in analyses further adjusted for influenza epidemics.

This study has limitations. First, we used monitoring data for ozone levels from fixed sites as a proxy for population exposure. Although this exposure assignment method is widely used in air pollution epidemiology studies (especially those exploring the effects of short-term exposure to air pollution) and ozone levels are spatially homogeneous compared with the levels of other air pollutants [38], it reportedly produces the Berkson-type measurement error, leading to lower precision rather than to bias [39]. Second, outcome misclassification is a concern. However, because we defined the outcome based on the principal diagnosis of hospital admission due to pneumonia determined by a physician, problems related to the precision of the diagnosis can be assumed to be low. In contrast, as we only analyzed relatively severe cases leading to hospital admission, it remains possible that the association between ozone and pneumonia would be attenuated after including relatively mild cases. Third, although negative control outcomes can be used to detect bias and strengthen causal inference using an observational study design [40], we could not apply them (e.g., hospital admissions due to appendicitis) in the present study, due to the lack of sufficient information. Fourth, this study used all hospitalization cases, rather than hospitalizations only through the emergency room, as an outcome; therefore, a considerable proportion of hospitalizations might have been scheduled and not associated with ozone exposure, leading the results toward the null hypothesis.

However, the present study has strengths that merit highlighting. First, this is one of a few studies that systematically evaluated the susceptible periods to ozone exposure regarding pneumonia among children and adolescents, who are considered vulnerable populations. Therefore, this study provides valuable evidence to select priority target populations for public health interventions and policies. Second, because the present study included all cases of hospital admissions due to pneumonia among children and adolescents in Korea during the study period (2011-2015), the possibility of selection bias is low. Third, to the best of our knowledge, this is the first study to apply a formal causal inference method to investigate the effect of ozone levels on pneumonia. Using a generalized difference-in-differences method, we could control for a wide range of confounders related to the spatial and temporal units of the study, thus substantially improving causal inference. Fourth, we assessed the independent effect of ozone on pneumonia by considering other air pollutants that can act as confounders. These analyses not only provide stronger evidence for a causal relationship, but also have direct public health implications, by aiding the formulation of air pollution standards for a specific air pollutant.

In conclusion, ozone levels were associated with a higher risk of pneumonia at 0-4 years and 5-9 years of age, but not at 10-14 years or 15-19 years of age. This study provides valuable insights to help identify vulnerable periods to ozone exposure in terms of pneumonia, determine the target populations for public health interventions and policies, and establish air pollution standards (considering the observed association between ozone and pneumonia below the current ozone standards). However, because the results of the present study might also reflect differences in outdoor activity patterns by age groups as well as vulnerability, future studies involving a more precise estimation of ozone exposure considering behavioral factors are needed.

## Figures and Tables

**Figure f2-epih-44-e2022002:**
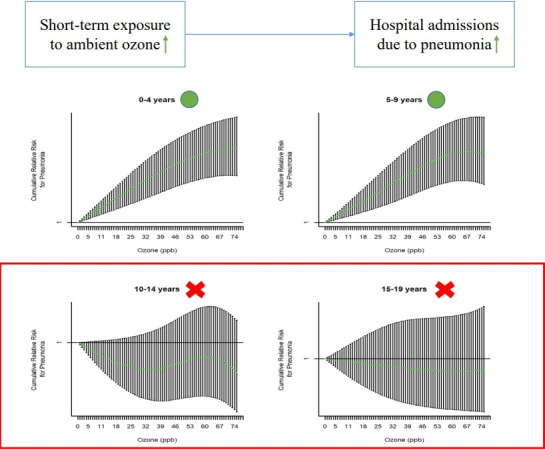


**Table 1.** Daily hospital admissions due to pneumonia, air pollution, and meteorological factors in Korea, 2011-2015

Variables	Mean±SD	Percentile					IQR
		Min	25th	50th	75th	Max	
Age (yr)							
0-4	493±276	88	295	424	633	2,507	338
5-9	100±90	6	46	70	114	726	68
10-14	29±26	0	12	21	35	274	23
15-19	12±10	0	6	10	15	117	9
Air pollution levels							
Ozone (ppb)^1^	37.3±13.4	6.8	26.8	36.5	46.7	90.3	19.9
PM_10_ (µg/m^3^)^2^	46.4±17.4	11.6	34.4	43.6	54.9	203.0	20.6
NO_2_ (ppb)^2^	20.7±8.9	2.5	14.2	19.2	25.7	63.3	11.6
SO_2_ (ppb)^2^	4.8±2.0	1.0	3.3	4.6	6.0	17.3	2.6
CO (ppb)^2^	496.0±150.3	172.3	394.9	469.9	568.8	1,502.0	173.9
Meteorological factors^3^							
Temperature (°C)	13.2±9.8	-14.6	4.8	14.4	21.7	33.1	16.9
Relative humidity (%)	67.0±15.2	11.3	56.8	68.5	78.3	99.9	21.5

SD, standard deviation; IQR, interquartile range; PM_10_, particulate matter with an aerodynamic diameter ≤10 μm; NO_2_, nitrogen dioxide; SO_2_, sulfur dioxide; CO, carbon monoxide; ppb, parts per billion.

1 Distribution of 7-day moving averages of daily 8-hour maximum concentrations during the study period (2011-2015).

2 Distribution of 7-day moving averages of daily mean concentrations during the study period (2011-2015).

3 Distribution of daily averages during the study period (2011-2015).

**Table 2.** Cumulative effects of ozone levels up to 7 days on pneumonia^1^, stratified by age group and seasons

Age (yr)	Other air pollutants^2^		Season	
	Crude	Adjusted	Warm (from May to October)	Cool (from November to April)
0-4	1.02 (1.01, 1.03)	1.02 (1.01, 1.03)	1.01 (0.99, 1.02)	1.06 (1.04, 1.09)
5-9	1.06 (1.04, 1.08)	1.06 (1.04, 1.08)	1.05 (1.02, 1.07)	1.07 (1.03, 1.12)
10-14	1.02 (0.99, 1.05)	1.01 (0.98, 1.04)	1.03 (0.99, 1.07)	0.95 (0.88, 1.02)
15-19	1.01 (0.97, 1.06)	1.01 (0.97, 1.06)	1.01 (0.96, 1.07)	1.09 (0.98, 1.20)

Values are presented as relative risk (95% confidence interval). ppb, parts per billion; PM_10_, particulate matter with an aerodynamic diameter ≤10 μm; NO_2_, nitrogen dioxide; SO_2_, sulfur dioxide; CO, carbon monoxide.

1 Cumulative effects were estimated for a 10.0-ppb increase in ozone levels, after adjustment for region, day, temperature, relative humidity, and population (and PM_10_, NO_2_, SO_2_, and CO in models further adjusted for other air pollutants).

2 PM_10_, NO_2_, SO_2_, and CO.

## SUPPLEMENTARY MATERIALS

Supplementary Material 1. Daily counts of a hospital admission due to pneumonia, 8-hour maximum ozone levels, and meteorological factors in 16 regions constituting the Republic of Korea, 2011–2015

Supplementary Material 2. The lag-specific associations between ozone levels and pneumonia by age group.

Supplementary Material 3. Daily counts of hospital admissions due to pneumonia, air pollution levels, and meteorological factors in the Republic of Korea, 2011–2015, stratified by season and sex

Supplementary Material 4. Correlation matrix for the correlations among air pollutant levels and meteorological factors.

Supplementary Material 5. Cumulative effects^a^ of PM_10_, NO_2_, SO_2_, and CO levels up to 7 days on pneumonia, estimated from distributed lag linear models

Supplementary Material 6. Cumulative effects of ozone levels up to 7 days on pneumonia^a^, stratified by sex

Supplementary Material 7. The associations between ozone levels and pneumonia by age group, excluding the region with the lowest ozone levels (Seoul).

Supplementary Material 8. The associations between ozone levels and pneumonia by age group, excluding the region with the highest ozone levels (Jeju-do).

Supplementary Material 9. The associations between ozone levels and pneumonia by age group, further adjusted for influenza epidemics.

Supplementary Material 10. The association between ozone levels and pneumonia by age group, estimated from conventional two-stage time-series analyses^a^
