# Supplementary material for: Age-specific effects of ozone on pneumonia in Korean children and adolescents: a nationwide time-series study
Source: Epidemiol Health. 2021 Dec 28;44:e2022002. doi: 10.4178/epih.e2022002 (PMC8989473; doi:10.4178/epih.e2022002)
Supplement: Supplementary Material 1. — Daily counts of a hospital admission due to pneumonia, 8-hour maximum ozone levels, and meteorological factors in 16 regions constituting the Republic of Korea, 2011–2015 [file epih-44-e2022002-suppl1.docx]

**Supplementary Material 1.** Daily counts of a hospital admission due to pneumonia, 8-hour maximum ozone levels, and meteorological factors in 16 regions constituting the Republic of Korea, 2011–2015

| Regions | Area (km^2^) | Population | Pneumonia^a^ | Ozone^a^ (ppb) | Temperature^a^ (℃) | Humidity^a^ (%) |
| --- | --- | --- | --- | --- | --- | --- |
| Seoul | 605 | 9,673,936 | 58.4 (38.0) | 28.1 (15.7) | 12.8 (10.9) | 59.8 (15.0) |
| Busan | 770 | 3,395,278 | 56.1 (34.7) | 34.9 (13.2) | 15.0 (8.4) | 61.8 (18.4) |
| Daegu | 884 | 2,444,412 | 33.4 (21.7) | 37.9 (19.2) | 14.5 (9.7) | 59.0 (17.3) |
| Incheon | 1,063 | 2,936,117 | 23.6 (15.9) | 34.5 (15.4) | 11.6 (10.0) | 71.5 (14.7) |
| Gwangju | 501 | 1,490,092 | 45.3 (20.3) | 36.7 (16.0) | 14.2 (9.7) | 67.2 (13.5) |
| Daejeon | 539 | 1,511,214 | 17.8 (11.3) | 34.2 (17.3) | 13.2 (10.3) | 69.5 (14.7) |
| Ulsan | 1,061 | 1,150,116 | 18.8 (15.1) | 36.4 (15.1) | 14.4 (9.0) | 63.6 (17.3) |
| Gyeonggi-do | 10,187 | 13,103,188 | 96.6 (63.9) | 35.9 (18.1) | 11.8 (10.8) | 67.3 (13.3) |
| Gangwon-do | 16,828 | 1,520,391 | 14.8 (12.1) | 40.2 (15.4) | 11.2 (10.2) | 66.1 (14.7) |
| Chungcheongbuk-do | 7,408 | 1,620,935 | 22.9 (15.2) | 38.7 (19.3) | 11.7 (10.5) | 66.7 (13.1) |
| Chungcheongnam-do | 8,691 | 2,493,790 | 25.4 (18.0) | 37.5 (15.6) | 12.3 (10.1) | 72.2 (11.9) |
| Jeollabuk-do | 8,069 | 1,818,157 | 29.8 (18.5) | 38.0 (15.6) | 12.7 (10.0) | 71.8 (11.4) |
| Jeollanam-do | 12,335 | 1,790,352 | 50.0 (26.1) | 38.6 (13.6) | 13.6 (9.0) | 72.5 (12.5) |
| Gyeongsangbuk-do | 19,033 | 2,672,902 | 36.0 (24.4) | 41.3 (15.2) | 12.4 (9.6) | 65.5 (15.0) |
| Gyeongsangnam-do | 10,540 | 3,350,350 | 93.4 (57.1) | 40.8 (16.6) | 13.8 (9.2) | 65.5 (14.9) |
| Jeju-do | 1,850 | 658,282 | 10.6 (7.7) | 41.6 (13.5) | 16.1 (7.6) | 72.7 (12.8) |

^a^Values are presented as mean (standard deviation).
