# Supplementary material for: Age-specific effects of ozone on pneumonia in Korean children and adolescents: a nationwide time-series study
Source: Epidemiol Health. 2021 Dec 28;44:e2022002. doi: 10.4178/epih.e2022002 (PMC8989473; doi:10.4178/epih.e2022002)
Supplement: Supplementary Material 3. — Daily counts of hospital admissions due to pneumonia, air pollution levels, and meteorological factors in the Republic of Korea, 2011–2015, stratified by season and sex [file epih-44-e2022002-suppl3.docx]

**Supplementary Material 3.** Daily counts of hospital admissions due to pneumonia, air pollution levels, and meteorological factors in the Republic of Korea, 2011–2015, stratified by season and sex

|  | Mean (SD) |  | Percentile | | | | |  | IQR |
| --- | --- | --- | --- | --- | --- | --- | --- | --- | --- |
|  |  |  | Min | 25th | 50th | 75th | Max |  |  |
| Daily counts of hospital admissions due to pneumonia | | | | | | | | | |
| Warm seasons (from May to October) | | | | | | | | | |
| 0–4 years | 451 (233) |  | 88 | 274 | 394 | 583 | 1,618 |  | 309 |
| 5–9 years | 99 (95) |  | 6 | 39 | 61 | 112 | 585 |  | 73 |
| 10–14 years | 26 (25) |  | 0 | 10 | 18 | 32 | 172 |  | 22 |
| 15–19 years | 12 (10) |  | 0 | 5 | 9 | 15 | 67 |  | 10 |
| Cool seasons (from November to April) | | | | | | | | | |
| 0–4 years | 536 (310) |  | 79 | 314 | 455 | 694 | 2,507 |  | 380 |
| 5–9 years | 102 (86) |  | 8 | 54 | 76 | 116 | 726 |  | 62 |
| 10–14 years | 31 (26) |  | 1 | 15 | 24 | 38 | 274 |  | 23 |
| 15–19 years | 13 (11) |  | 0 | 6 | 10 | 16 | 117 |  | 10 |
| Boys |  |  |  |  |  |  |  |  |  |
| 0–4 years | 264 (146) |  | 46 | 159 | 227 | 340 | 1,368 |  | 181 |
| 5–9 years | 51 (46) |  | 0 | 23 | 36 | 59 | 397 |  | 36 |
| 10–14 years | 14 (13) |  | 0 | 6 | 11 | 18 | 142 |  | 12 |
| 15–19 years | 6 (5) |  | 0 | 3 | 5 | 8 | 58 |  | 5 |
| Girls |  |  |  |  |  |  |  |  |  |
| 0–4 years | 228 (131) |  | 30 | 134 | 196 | 294 | 1,139 |  | 160 |
| 5–9 years | 49 (45) |  | 2 | 22 | 34 | 56 | 353 |  | 34 |
| 10–14 years | 15 (13) |  | 0 | 6 | 10 | 18 | 133 |  | 12 |
| 15–19 years | 6 (6) |  | 0 | 2 | 5 | 8 | 59 |  | 6 |
|  |  |  |  |  |  |  |  |  |  |
| Air pollution levels | | | | | | | | | |
| Warm seasons (from May to October) | | | | | | | | | |
| Ozone^a^ (ppb) | 43.0 (16.6) |  | 4.1 | 31.0 | 41.9 | 54.4 | 110.7 |  | 23.3 |
| PM_10_^b^ (µg/m^3^) | 40.8 (23.4) |  | 4.3 | 26.7 | 36.3 | 49.5 | 598.8 |  | 22.8 |
| NO_2_^b^ (ppb) | 17.6 (9.1) |  | 1.2 | 11.2 | 15.4 | 21.8 | 80.2 |  | 10.6 |
| SO_2_^b^ (ppb) | 4.1 (2.2) |  | 0.7 | 2.6 | 3.7 | 5.0 | 25.7 |  | 2.4 |
| CO^b^ (ppb) | 412.1 (111.3) |  | 132.3 | 334.7 | 400.6 | 478.2 | 1,093.5 |  | 143.5 |
| Cool seasons (from November to April) | | | | | | | | | |
| Ozone^c^ (ppb) | 31.4 (13.9) |  | 1.5 | 22.1 | 29.7 | 39.4 | 92.8 |  | 17.3 |
| PM_10_^d^ (µg/m^3^) | 52.1 (26.8) |  | 6.0 | 34.6 | 47.0 | 63.3 | 555.7 |  | 28.6 |
| NO_2_^d^ (ppb) | 23.8 (11.3) |  | 0.8 | 15.5 | 21.7 | 29.8 | 82.5 |  | 14.3 |
| SO_2_^d^ (ppb) | 5.5 (2.3) |  | 0.7 | 4.0 | 5.1 | 6.6 | 24.3 |  | 2.7 |
| CO^d^ (ppb) | 582.9 (206.6) |  | 133.8 | 436.8 | 539.0 | 682.7 | 1,983.3 |  | 245.9 |
|  |  |  |  |  |  |  |  |  |  |
| Meteorological factors | | | | | | | | | |
| Warm seasons (from May to October)^b^ | | | | | | | | | |
| Temperature (℃) | 21.3 (4.5) |  | 3.7 | 18.3 | 21.7 | 24.6 | 33.1 |  | 6.3 |
| Relative humidity (%) | 72.7 (12.5) |  | 20.4 | 65.0 | 73.8 | 81.7 | 99.9 |  | 16.8 |
| Cool seasons (from November to April)^d^ | | | | | | | | | |
| Temperature (℃) | 5.0 (6.3) |  | -14.5 | 0.5 | 4.8 | 9.9 | 22.9 |  | 9.4 |
| Relative humidity (%) | 61.3 (15.4) |  | 11.3 | 50.2 | 61.4 | 72.2 | 99.9 |  | 22.0 |

Abbreviations: SD, standard deviation; IQR, interquartile range

^a^Distribution of daily 8-hour maximum concentrations during warm seasons of the study period (2011–2015).

^b^Distribution of daily mean concentrations during warm seasons of the study period (2011–2015).

^c^Distribution of daily 8-hour maximum concentrations during cool seasons of the study period (2011–2015).

^d^Distribution of daily mean concentrations during cool seasons of the study period (2011–2015).
