# Supplementary material for: Age-specific effects of ozone on pneumonia in Korean children and adolescents: a nationwide time-series study
Source: Epidemiol Health. 2021 Dec 28;44:e2022002. doi: 10.4178/epih.e2022002 (PMC8989473; doi:10.4178/epih.e2022002)
Supplement: Supplementary Material 5. — Cumulative effectsa of PM10, NO2, SO2, and CO levels up to 7 days on pneumonia, estimated from distributed lag linear models [file epih-44-e2022002-suppl5.docx]

**Supplementary Material 5.** Cumulative effects^a^ of PM_10_, NO_2_, SO_2_, and CO levels up to 7 days on pneumonia, estimated from distributed lag linear models

|  | PM_10_ | | |  | NO_2_ | | |  | SO_2_ | | |  | CO | | |
| --- | --- | --- | --- | --- | --- | --- | --- | --- | --- | --- | --- | --- | --- | --- | --- |
|  | Single^b^ |  | Multi^c^ |  | Single^b^ |  | Multi^c^ |  | Single^b^ |  | Multi^c^ |  | Single^b^ |  | Multi^c^ |
|  | RR  (95% CI) |  | RR  (95% CI) |  | RR  (95% CI) |  | RR  (95% CI) |  | RR  (95% CI) |  | RR  (95% CI) |  | RR  (95% CI) |  | RR  (95% CI) |
| 0–4 years | 1.01  (1.01, 1.02) |  | 1.01  (1.00, 1.02) |  | 1.01  (1.00, 1.02) |  | 1.00  (0.99, 1.02) |  | 1.01  (1.00, 1.01) |  | 1.01  (1.00, 1.01) |  | 1.01  (1.00, 1.02) |  | 0.99  (0.98, 1.01) |
| 5–9 years | 1.01  (1.00, 1.02) |  | 0.99  (0.98, 1.01) |  | 0.99  (0.97, 1.02) |  | 0.96  (0.93, 0.99) |  | 1.00  (0.99, 1.01) |  | 0.99  (0.98, 1.00) |  | 1.10  (1.07, 1.13) |  | 1.15  (1.12, 1.19) |
| 10–14 years | 1.01  (0.99, 1.03) |  | 1.01  (0.99, 1.04) |  | 0.96  (0.92, 1.00) |  | 0.91  (0.87, 0.95) |  | 0.98  (0.97, 1.00) |  | 0.97  (0.95, 0.98) |  | 1.10  (1.06, 1.14) |  | 1.18  (1.13, 1.24) |
| 15–19 years | 1.00  (0.98, 1.03) |  | 1.00  (0.97, 1.03) |  | 0.99  (0.93, 1.04) |  | 0.96  (0.90, 1.03) |  | 0.98  (0.96, 1.00) |  | 0.96  (0.94, 0.98) |  | 1.08  (1.03, 1.14) |  | 1.16  (1.09, 1.24) |

Abbreviations: RR, relative risk; CI, confidence interval

^a^Results are presented by 10.0 ppb increment for NO_2_, 10.0 µg/m^3^ increment for PM_10_, 1.0 ppb increment for SO_2_, and 0.2 ppm increment for CO.

^b^Estimated using quasi-Poisson regression models adjusted for region, day, temperature, relative humidity, and population.

^c^Estimated using quasi-Poisson regression models adjusted for region, day, temperature, relative humidity, and population and with terms for air pollutants (ozone, PM_10_, NO_2_, SO_2_, and CO).
