# Supplementary material for: Age-specific effects of ozone on pneumonia in Korean children and adolescents: a nationwide time-series study
Source: Epidemiol Health. 2021 Dec 28;44:e2022002. doi: 10.4178/epih.e2022002 (PMC8989473; doi:10.4178/epih.e2022002)
Supplement: Supplementary Material 6. — Cumulative effects of ozone levels up to 7 days on pneumoniaa, stratified by sex [file epih-44-e2022002-suppl6.docx]

**Supplementary Material 6.** Cumulative effects of ozone levels up to 7 days on pneumonia^a^, stratified by sex

|  | Boys | |  | Girls | |
| --- | --- | --- | --- | --- | --- |
|  | RR | 95% CI |  | RR | 95% CI |
| 0–4 years | 1.02 | (1.01, 1.03) |  | 1.03 | (1.02, 1.04) |
| 5–9 years | 1.05 | (1.02, 1.07) |  | 1.07 | (1.04, 1.10) |
| 10–14 years | 1.01 | (0.98, 1.06) |  | 1.00 | (0.96, 1.04) |
| 15–19 years | 1.04 | (0.97, 1.10) |  | 0.98 | (0.93, 1.05) |

Abbreviations: RR, relative risk; CI, confidence interval

^a^Cumulative effects were estimated for a 10.0-ppb increase in ozone levels, after adjustment for region, day, temperature, relative humidity, population, and other air pollutants (PM_10_, NO_2_, SO_2_, and CO).
