# Supplementary material for: Age-specific effects of ozone on pneumonia in Korean children and adolescents: a nationwide time-series study
Source: Epidemiol Health. 2021 Dec 28;44:e2022002. doi: 10.4178/epih.e2022002 (PMC8989473; doi:10.4178/epih.e2022002)
Supplement: Supplementary Material 9. — The associations between ozone levels and pneumonia by age group, further adjusted for influenza epidemics. [file epih-44-e2022002-suppl9.docx]

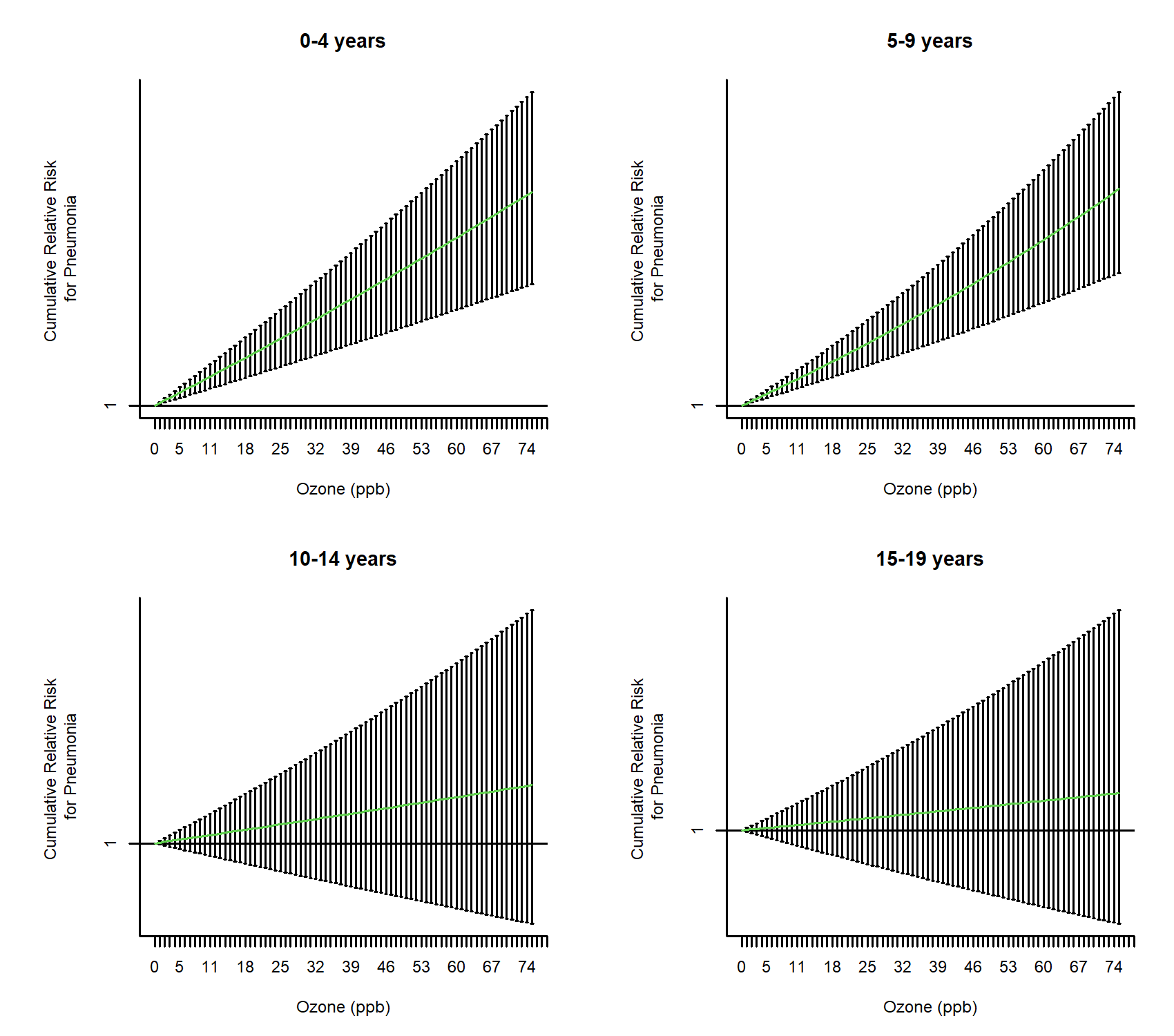


**Supplementary Material 9.** The associations between ozone levels and pneumonia by age group, further adjusted for influenza epidemics.
