# Supplementary material for: Age-specific effects of ozone on pneumonia in Korean children and adolescents: a nationwide time-series study
Source: Epidemiol Health. 2021 Dec 28;44:e2022002. doi: 10.4178/epih.e2022002 (PMC8989473; doi:10.4178/epih.e2022002)
Supplement: Supplementary Material 10. — The association between ozone levels and pneumonia by age group, estimated from conventional two-stage time-series analysesa [file epih-44-e2022002-suppl10.docx]

**Supplementary Material 10.** The association between ozone levels and pneumonia by age group, estimated from conventional two-stage time-series analyses^a^

|  | Fixed effect model^b^ | |  | Random effect model^b^ | |
| --- | --- | --- | --- | --- | --- |
|  | RR | 95% CI |  | RR | 95% CI |
| 0–4 years | 1.01 | (1.01, 1.02) |  | 1.01 | (1.01, 1.02) |
| 5–9 years | 1.00 | (0.99, 1.01) |  | 1.00 | (0.99, 1.01) |
| 10–14 years | 0.99 | (0.98, 1.01) |  | 0.99 | (0.97, 1.01) |
| 15–19 years | 1.01 | (0.99, 1.03) |  | 1.01 | (0.99, 1.03) |

Abbreviations: RR, relative risk; CI, confidence interval

^a^The results are presented for a 10.0-ppb increase in ozone levels. The time-series models were adjusted for calendar time (natural cubic spline, 8 df per year), daily mean temperature and relative humidity up to 7 days (cross-basis matrices, 3 df), and other pollutants (PM_10_, NO_2_, SO_2_, and CO) up to 7 days (cross-basis matrices).

^b^*p*-values for a heterogeneity test were 0.92 at 0–4 years of age, 0.003 at 5–9 years of age, 0.10 at 10–14 years of age, and 0.34 at 15–19 years of age.
